# Family-Based Interpretation of a Prenatally Detected 15q11.2 Duplication

**DOI:** 10.7759/cureus.103945

**Published:** 2026-02-20

**Authors:** Andreas E Spathi, Stylianos Lagios, Vassilis Papanikolaou, Charilaos Kostoulas, Athanasia Sesse, Eleni Theochari, Theoni Leoutsakou, Alexandros Psarris, Maria Kavvadia, Theano Stauroulaki, Aspasia Destouni, Ioannis Georgiou, Andreas Pampanos

**Affiliations:** 1 Department of Genetics, Alexandra General Hospital, Athens, GRC; 2 Laboratory of Medical Genetics, Faculty of Medicine, School of Health Sciences, University of Ioannina, Ioannina, GRC; 3 First Department of Obstetrics and Gynecology, Alexandra General Hospital, National and Kapodistrian University of Athens, Athens, GRC; 4 Third Department of Obstetrics and Gynecology, School of Medicine, Aristotle University of Thessaloniki, Thessaloniki, GRC

**Keywords:** 15q11.2 duplication, array cgh, family studies, genetic counselling, prenatal testing

## Abstract

Chromosome region 15q11-q13 is prone to structural rearrangements and contains imprinted genes associated with several neurodevelopmental syndromes. In this report, we present the case of a 518 kb duplication in 15q11.2, identified prenatally through array comparative genomic hybridization. Parental testing revealed that the duplication was paternally inherited and originated from the asymptomatic paternal grandmother. The duplicated region harbored the OMIM genes *TUBGCP5*, *CYFIP1*, *NIPA1*, and *NIPA2*, all of which presented biallelic expression and were not subjected to genomic imprinting. Given the benign familial inheritance and lack of clinical features in the father and the paternal grandmother, the duplication was considered likely to have benign significance. A healthy female newborn was delivered at term.

## Introduction

Chromosome 15 is an acrocentric chromosome rich in low-copy repeats (LCRs), rendering it particularly unstable and susceptible to cytogenetic alterations such as deletions and duplications. In addition, it is among the chromosomes that harbor numerous imprinted genes. Notably, the 15q11-q13 region contains several imprinted loci associated with syndromes such as Prader-Willi syndrome and Angelman syndrome, which result from the loss of expression of paternally and maternally expressed genes, respectively. In addition, (micro)duplications or partial tetrasomy of the 15q11-q13 region give rise to the 15q11-q13 duplication syndrome (OMIM #608636), an autosomal dominant neurodevelopmental disorder characterized by variable expressivity and incomplete penetrance, whose phenotype may include features such as autism spectrum disorder (ASD), seizures, ataxia, behavioral issues, developmental delay, and dysmorphic features [1,2]. Within this region, duplication of the 15q11.2 BP1-BP2 interval represents an emerging copy number variation (CNV) with low pathogenicity, incomplete penetrance, and variable expressivity, and it is commonly identified both in affected and unaffected individuals, complicating its clinical interpretation, especially in the prenatal setting. Reported phenotypes among duplication carriers include developmental, motor, and language delay, ASD, behavioral problems, epilepsy, and hypotonia. However, its high prevalence in unaffected populations supports a minimal increase in risk for abnormal neurodevelopmental outcomes, despite encompassing *TUBGCP5*, *CYFIP1*, *NIPA1*,* *and *NIPA2*, evolutionarily conserved genes expressed in the central nervous system and implicated in neurodevelopmental pathways. Prenatal genetic counseling is therefore challenging due to limited and sometimes conflicting evidence, variable expressivity, and incomplete penetrance, particularly when ultrasound abnormalities are present, while recent studies advise caution in attributing pathogenic significance to this duplication, including its proposed associations with autism and schizophrenia [3-5]. This case report aims to emphasize the value of multigenerational family studies in the interpretation of prenatally identified 15q11.2 BP1-BP2 duplications.

## Case presentation

A couple (female, 31 years old; male, 35 years old) in an ongoing pregnancy sought consultation from the Department of Genetics, Alexandra Hospital, Greece. This pregnancy was the couple’s first, naturally conceived, and routine prenatal ultrasound examinations revealed fetal renal pelvic dilatation. Amniocentesis was performed at 16+6 weeks of pregnancy, and high-resolution molecular karyotyping was conducted at an external laboratory, revealing a duplication of 518 Kb in 15q11.2 (GRCh37: 15q11.2 (22770421-23288350)). They were referred to our laboratory for genetic counseling and further investigation. The initial strategy was to check the parents to conclude whether the finding occurred *de novo* or was of parental origin. Upon reviewing the existing literature, conflicting data regarding this finding were revealed, indicating variable expressivity and incomplete penetrance, with symptoms comprising different levels of neurological and developmental abnormalities, behavioral disorders within the context of the autistic spectrum, and speech deficits [6-8].

We further examined literature data considering whether genomic imprinting might be involved, based on the fact that many regions of chromosome 15 are imprinted. This initial research through Decipher sorting revealed a possible implication of maternal inheritance to pathogenic phenotypes, while paternal duplications were mainly detected in healthy individuals (deciphergenomics.org).

**Figure 1:**
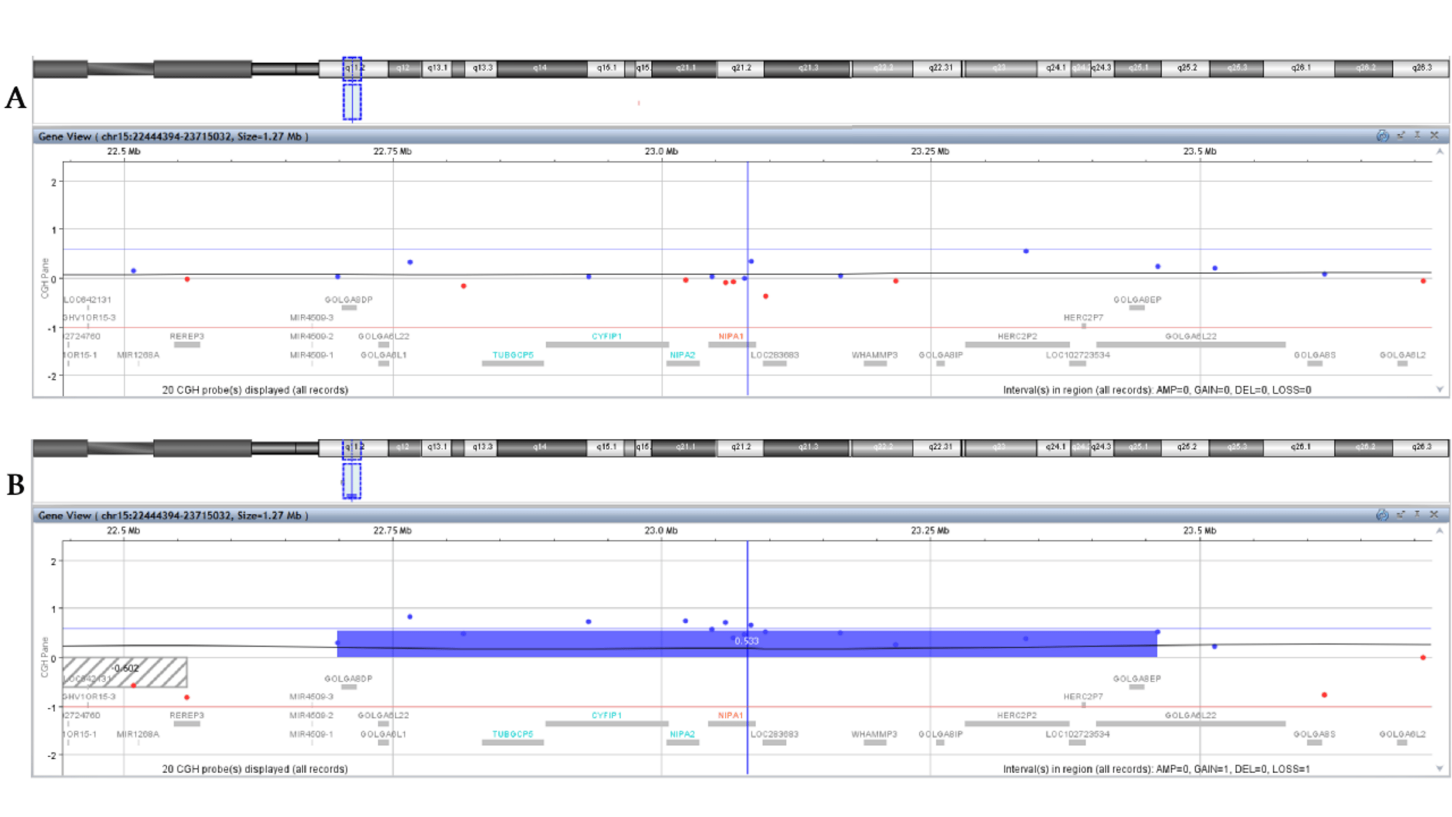
Microarray comparative genomic hybridization results for the 15q11.2 region from 22444394 to 23715032, including the chr15:22770421-23288350 duplicated region found in the embryo, for the embryo’s mother (A) and father (B). Each dot represents a different probe, blue dots for regions overrepresented in the sample compared to the reference, and red dots for regions underrepresented in the sample compared to the reference. The duplicated region is highlighted with the blue bar. As illustrated, the embryo’s duplication was detected in the father’s sample (B).

**Figure 2:**
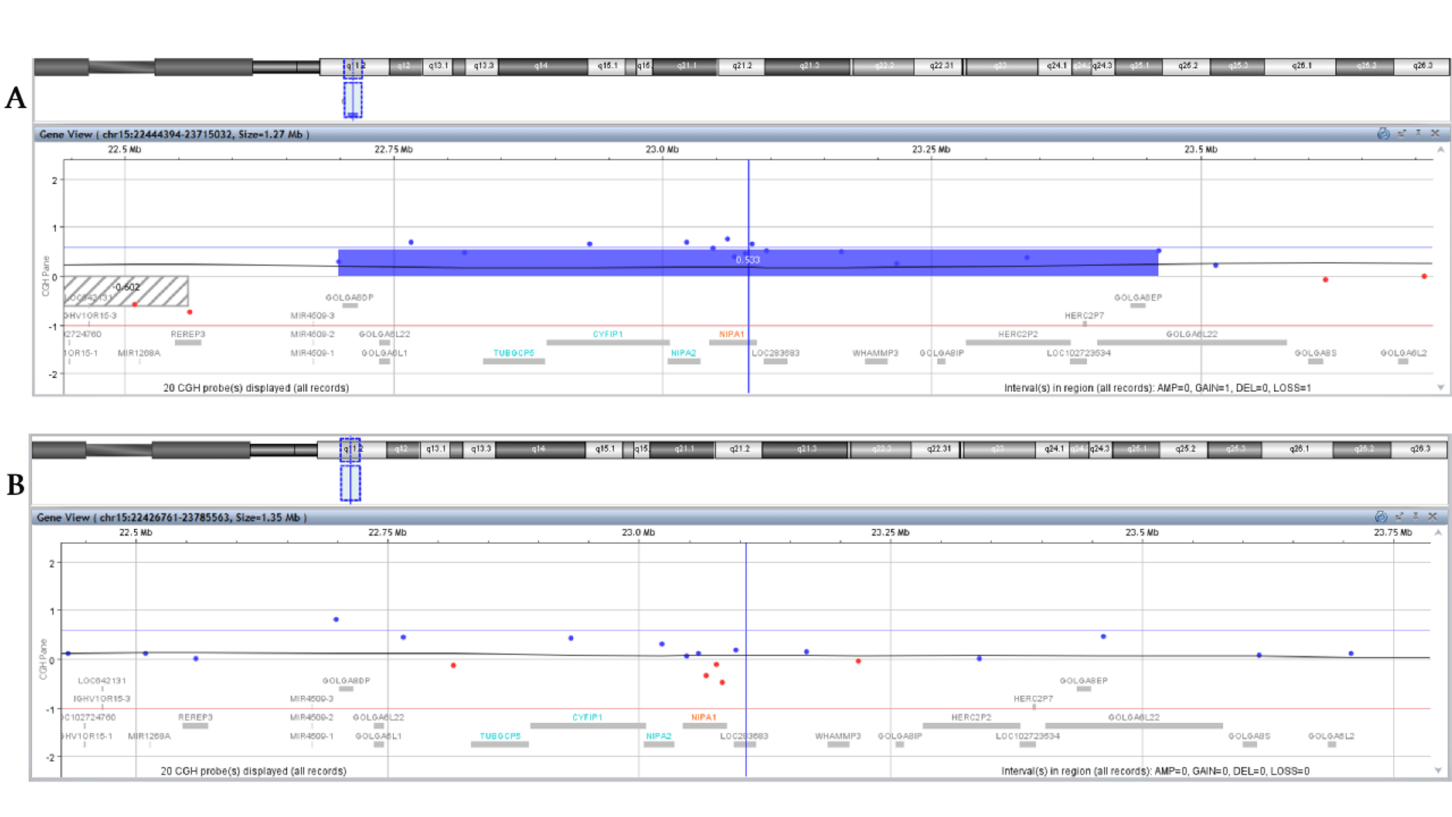
Comparative genomic hybridization results for the 15q11.2 region from 22444394 to 23715032, including the chr15:22770421-23288350 duplicated region found in the embryo, for the embryo’s paternal grandmother (A) and paternal grandfather (B). Each dot represents a different probe, blue dots for regions overrepresented in the sample compared to the reference and red dots for regions underrepresented in the sample compared to the reference, and the duplicated region is highlighted with the blue bar. As illustrated, the embryo’s duplication was detected in the paternal grandmother’s sample (A).

**Figure 3:**
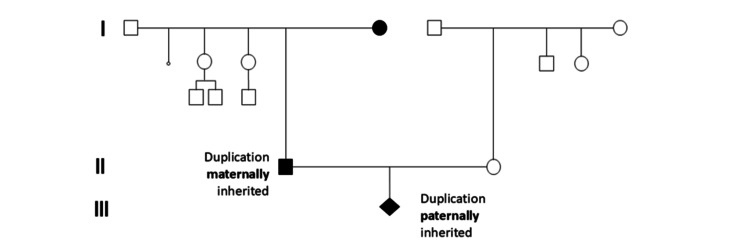
Family pedigree depicting the inheritance of the 15q11.2 duplication. Family pedigree depicting the inheritance of the 15q11.2 duplication after microarray comparative genomic hybridization was performed in samples from three generations.

Prenatal testing was conducted in another laboratory using the Affymetrix Cytogenetics Whole-Genome Cytoskan 750K array (Thermo Fisher Scientific, Waltham, MA, USA). In our laboratory, parental peripheral blood samples were collected in EDTA tubes, and DNA was extracted using the Maxwell® 16 Blood Purification Kit (Promega, Madison, WI, USA). Microarray comparative genomic hybridization (a-CGH) was performed using the GenetiSure Cyto 8x60K platform (Agilent Technologies, Santa Clara, CA, USA), an assay that is able to detect the 15q11.2 duplication, even if the probes used are different and differently spaced. The results were analyzed with Cytogenomics software version 5.1.2.1 (Agilent Technologies, Santa Clara, CA, USA) using Build 37 (UCSC GRCh37, 2009) from the National Center for Biotechnology Information as the reference genome.

a-CGH analysis of the parents revealed a normal genetic profile for the mother, with no evidence of clinically relevant chromosome CNVs in the analyzed regions, including the region of interest, 15q11.2 (Figure 1A). In contrast, the father was found to carry a 762 kb duplication in 15q11.2 (GRCh37: 15q11.2 (22698522-23460905)) (Figure 1B). The difference in the size of the detected duplication compared to the 518 kb detected in the embryo’s DNA is due to the different platform used, but does not alter the result, neither increases the number of implicated genes. This initial finding, together with the literature findings that paternal-originated duplications seemed to lead to healthy descendants, was an initial comforting finding for the parents during genetic counseling. Prompted by this finding, the parents wished to proceed with testing of the paternal grandparents. As the father was asymptomatic, and according to the literature data, we would have expected to detect the 15q11.2 duplication in the grandfather. However, as illustrated in Figure 2A and Figure 2B, it was not the grandfather but the grandmother who was found to harbor the 15q11.2 duplication. A three-generation pedigree was constructed to assess inheritance patterns (Figure 3).

## Discussion

The 15q11-q13 microduplication syndrome has been associated with ASD, intellectual disability, seizures, and psychiatric conditions such as schizophrenia [9-11]. Furthermore, current evidence suggests a parent-of-origin effect, as maternally inherited duplications of the 15q11-q13 region are up to now more frequently associated with pathological phenotypes compared to paternally inherited ones [12,13].

Using Decipher Genomics (deciphergenomics.org), we identified the genes located within this region: *TUBGCP5*, *CYFIP1*, *NIPA2*, *NIPA1*, *GOLGA8DP*, *GOLGA6L22*, *LOC283683*, *WHAMMP3*, *GOLGA8IP*, *HERC2P2*, *LOC102723534*, *HERC2P7*, and *GOLGA8EP*. Among these, we focused on the coding genes *TUBGCP5*, *CYFIP1*, *NIPA2*, and *NIPA1*, which are registered in OMIM and predicted to exhibit triplosensitivity. According to ClinGen, duplication of the 15q11.2 (BP1-BP2) region is currently considered to lack established clinical significance, as it represents a common CNV in the general population. Consequently, this region’s triplosensitivity score is classified as “dosage sensitivity unlikely” (Score 40) [14-16], and duplication carriers appear to perform similarly to non-carriers on cognitive assessments [17]. The overall prevalence of 15q11.2 BP1-BP2 CNVs has been estimated at approximately 0.5-1% in the general population, while Chu et al. reported a prevalence of 1.5%, comprising 0.7% microdeletions and 0.8% microduplications in this region [4,5]. The four coding genes within the region of interest are evolutionarily conserved, expressed in the central nervous system, and implicated in axonal growth and neural connectivity [18]. Starting with *TUBGCP5 *(OMIM *608147), it encodes for tubulin gamma complex component 5, which enables microtubule activity, is involved in microtubule nucleation, and has been associated with neurodevelopmental disorders such as attention-deficit/hyperactivity disorder and obsessive-compulsive disorder (OCD) [18-20]. *CYFPI1 *(OMIM *606322) translates to a protein regulating the cytoskeleton’s dynamics, impacting aspects of dendritic spine formation, stability, morphology, migration, and excitability of neural cells, and protein translation, rendering it important for synaptic plasticity. Thus, it is a gene implicated in intellectual disability, ASD, schizophrenia, and epilepsy [21-23]. Finally, *NIPA1 *(OMIM *608145) and *NIPA2 *(OMIM *608146) encode magnesium transporters that are involved in neuronal magnesium homeostasis [24,25]. *NIPA1 *variants cause hereditary spastic paraplegia type 6 (OMIM #600363) [26,27], while NIPA2 alterations have been implicated, though inconsistently, in childhood absence epilepsy [28-30]. Both *NIPA1* and *NIPA2*, along with *TUBGCP5 *and *CYFPI1*, exhibit biallelic expression, meaning they are not subject to genomic imprinting [24,31-33]. Consequently, the phenotypic outcome of a duplication involving these genes does not depend on the parental origin of the inherited variant.

Considering the above findings, genetic counseling was provided to the couple based on the a-CGH results of the family members (Table 1) in conjunction with current literature data. The detection of both maternally and paternally inherited 15q11.2 BP1-BP2 duplications in two generations of healthy individuals (Table 1), along with evidence suggesting that the four genes within this region are not subject to genomic imprinting, supports the interpretation that, at least in this Greek family, the duplication is not currently associated with a pathogenic phenotype.

**Table 1:** Summary of the aCGH findings from three generations. aCGH = microarray comparative genomic hybridization; CNV = copy number variation

Generation	Family member	CNV size	Coordinates (GRCh37)	Inheritance pattern	Phenotype
III	Proband	518 kb duplication	chr15:22770421-23288350	Paternally inherited	Unaffected
II	Mother	-	-	-	Unaffected
	Father	762 kb duplication	chr15:22698522-23460905	Maternally inherited	Unaffected
I	Paternal grandmother	762 kb duplication	chr15:22698522-23460905	Unknown	Unaffected
	Paternal grandfather	-	-	-	Unaffected

It was recommended that first-degree paternal relatives of reproductive age undergo genetic testing and that prenatal testing should be performed in any future pregnancies of his spouse. Subsequent testing of the paternal grandparents revealed that the embryo’s father had inherited the duplication from his mother (the embryo’s paternal grandmother), confirming paternal inheritance of the duplication in the embryo. Because both the father and paternal grandmother are asymptomatic and do not exhibit features associated with the 15q11.2 BP1-BP2 pathogenic phenotype, the embryo was not expected to be clinically affected. Nevertheless, careful pregnancy monitoring through detailed ultrasonographic evaluation was recommended, as well as genetic testing of the father’s sisters to provide more comprehensive reproductive counseling. The pregnancy proceeded to term, and a healthy female newborn was delivered. At the 18-month postnatal follow-up, the child demonstrated normal growth and neurodevelopment, with no phenotypic features attributable to the 15q11.2 BP1-BP2 duplication. The previously observed fetal renal pelvic dilatation resolved spontaneously and was considered an incidental, unrelated finding to the observed duplication. It was advised that the identified 15q11.2 duplication be documented in the child’s medical record to ensure appropriate consideration in future genetic and reproductive counseling.

## Conclusions

The 15q11.2 (BP1-BP2) duplication identified prenatally was paternally inherited from two asymptomatic carriers, the father and the paternal grandmother. The four coding genes in this region are reported to exhibit biallelic expression, suggesting that parent-of-origin effects are unlikely to play a major role in phenotypic expression. Current evidence suggests that dup15q11.2 is a relatively common CNV in the general population. It is generally associated with low pathogenic potential, and the absence of clinical manifestations in this Greek family supports its interpretation as likely benign or of low clinical significance. Nevertheless, given the incomplete penetrance and variable expressivity associated with this CNV, genetic counseling remains important for future reproductive planning, and continued clinical follow-up of the child is appropriate to monitor developmental outcomes. This case report highlights the value of multigenerational family studies in improving the interpretation of CNVs detected during prenatal testing.
